# 

**Published:** 1831-07

**Authors:** 


### THE LONDON MEDICAL AND PHYSICAL JOURNAL.

EDITED BY

JOHN NORTH, ESQ. F. L. S.

MEMBER OF THE ROYAL COLLEGE OF SURGEONS,

AND OF THE MEDICAL AND CHIRURGICAL SOCIETY OF LONDON.

---

(VOL. LXVI.)

NEW SERIES, VOL. XI.

---

Et quoniam variant morbi, variabimus artes;

Mille mali species, mille salutis erunt.

---

45597

LONDON:

JOHN SOUTER, 73, ST. PAUL'S CHURCH-YARD;

AND TO BE HAD OF ALL MEDICAL BOOKSELLERS.

1831.

W1
LO 31

C. ADLARD, PRINTER, BARTHOLOMEW CLOSE.

*** *Communications have been received from* Mr. Russell, Medicus, *&c.*

